# Supplementary material for: Propagation of optically tunable coherent radiation in a gas of polar molecules
Source: Sci Rep. 2020 Oct 19;10:17615. doi: 10.1038/s41598-020-74569-w (PMC7572405; doi:10.1038/s41598-020-74569-w)
Supplement: Supplementary file 1 — Supplementary Information. [file 41598_2020_74569_MOESM1_ESM.pdf]

Propagation of optically tunable coherent radiation in a gas of polar molecules: supplementary information

Piotr Gładysz[1,*], Piotr Wcisło[1], and Karolina Słowik[1]

[1]Institute of Physics, Faculty of Physics, Astronomy and Informatics, Nicolaus Copernicus University in Toruń, Grudziadzka 5, 87-100 Torun, Poland
[*]glad@doktorant.umk.pl

ABSTRACT

This file contains the following supplementary information:
S1. Relation between broken inversion symmetry and dipole moment
S2. Derivation of the Bloch-Maxwell equations
S3. Details of the numerical approach
S4. Lithium hydride example

S1 Relation between broken inversion symmetry and dipole moment

A diagonal element of the dipole moment operator with $i = e, g$ is given by

$$\mathbf{d}_{ii} = \langle i|\,\hat{\mathbf{d}}\,|i\rangle = \int d\mathbf{r} \sum_{\alpha} q_\alpha \mathbf{r}_\alpha \langle i|\mathbf{r}\rangle \langle \mathbf{r}|i\rangle\,, \tag{S1}$$

where $\mathbf{r} = \{\mathbf{r}_\alpha\}_{\alpha=1,\dots,N}$ represents the set of positions $\mathbf{r}_\alpha$ of all N charges q_α contributing to the dipole moment of the system. For simplicity, in this proof we assume a single charge q at position $\mathbf{r}_1$. The proof for multiple charges is a straightforward generalization.

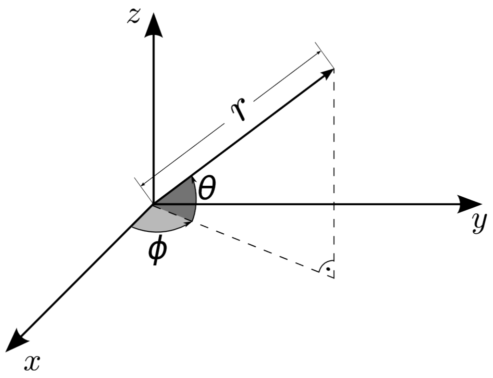

Figure S1. Spherical coordinate system used in calculations according to (S2). The unusual choice of directed angles θ and ϕ (notice the arrows in the picture) was intentional to set symmetric limits of integration in the equations.

To evaluate this quantity, we express $\mathbf{r}_1$ in spherical coordinates defined as follows (Fig. S1):

$$\begin{aligned} x &= r\cos\phi\cos\theta, \\ y &= r\sin\phi\cos\theta, \\ z &= r\sin\theta. \end{aligned} \tag{S2}$$

to obtain

$$\begin{aligned}\mathbf{d}_{ii} &= q\int_0^\infty dr_1 \int_{-\pi}^{\pi} d\phi_1 \int_{-\pi/2}^{\pi/2} d\theta_1\; r_1^2 \cos\theta_1\; \mathbf{r}_1\; |\langle \mathbf{r}_1|i\rangle|^2 \\ &= q\int_0^\infty dr_1 \int_{-\pi}^{\pi} d\phi_1 \int_{0}^{\pi/2} d\theta_1\; r_1^2 \cos\theta_1\; \mathbf{r}_1\; |\langle \mathbf{r}_1|i\rangle|^2 + q\int_0^\infty dr_1 \int_{-\pi}^{\pi} d\phi_1 \int_{-\pi/2}^{0} d\theta_1\; r_1^2 \cos\theta_1\; \mathbf{r}_1\; |\langle \mathbf{r}_1|i\rangle|^2\end{aligned} \tag{S3}$$

By substituting angles $\phi \to -\phi$, $\theta \to -\theta$ in the second term and making use of the relation $\cos(-\theta) = \cos(\theta)$, we obtain

$$\mathbf{d}_{ii} = q\int_0^\infty dr_1 \int_{-\pi}^{\pi} d\phi_1 \int_{0}^{\pi/2} d\theta_1\; r_1^2 \cos\theta_1\; \mathbf{r}_1 \left(|\langle \mathbf{r}_1|i\rangle|^2 - |\langle -\mathbf{r}_1|i\rangle|^2 \right), \tag{S4}$$

where $-\mathbf{r}_1 = (r_1, -\phi_1, -\theta_1)$. If the wave function $\langle \mathbf{r}_1|i\rangle$ has inversion symmetry, $|\langle \mathbf{r}_1|i\rangle| = |\langle -\mathbf{r}_1|i\rangle|$ holds, and the bracket at the end disappears. This implies that the diagonal elements d_{ee} and d_{gg} are equal to zero. This means that broken inversion symmetry is required to create a permanent dipole moment.

For the off-diagonal elements, we simply have

$$\mathbf{d}_{ij} = \langle i|\hat{\mathbf{d}}|j\rangle = \int \mathrm{d}\mathbf{r}\, \mathbf{r}\, \langle i|\mathbf{r}\rangle \langle \mathbf{r}|q\hat{\mathbf{r}}|\mathbf{r}_1\rangle \langle \mathbf{r}_1|j\rangle = \mathbf{d}_{ji}^{\star}, \tag{S5}$$

regardless of the wave function's symmetry. Due to the odd character of the position operator, if both wave functions share the same symmetry, the integral is equal to zero, and the transition is electric-dipole-forbidden.

S2 Bloch–Maxwell equations

Here, we provide a detailed derivation of the Bloch–Maxwell equations. First, let us insert the form of the field given by Eq. (1) into the master equation (6). The set of equations for the density matrix elements has the following form:

$$\mathrm{i}\hbar\frac{\partial}{\partial t}\rho_{ee} = \left(\mathscr{E}\cos(kz-\omega t) + E_{\text{signal}}\right)\left(d_{ge}\rho_{eg} - d_{eg}\rho_{ge}\right) - 2\mathrm{i}\hbar\gamma_{\text{se}}\rho_{ee}, \tag{S6a}$$

$$\mathrm{i}\hbar\frac{\partial}{\partial t}\rho_{eg} = \hbar\omega_0\rho_{eg} - \left(\mathscr{E}\cos(kz-\omega t) + E_{\text{signal}}\right)\left((d_{ee}-d_{gg})\rho_{eg} - d_{eg}(2\rho_{ee}-1)\right) - \mathrm{i}\hbar(\gamma_{\text{se}}+\gamma_{\text{dec}})\rho_{eg}. \tag{S6b}$$

The dynamics of the other elements can be found based on the density matrix properties: $\rho_{ee}+\rho_{gg} = 1$ and $r_{ge} = r_{eg}^{\star}$. Next, we make use of the ansatz (9) and expand the κ-dependent term into a series of Bessel functions $e^{-\mathrm{i}\kappa\sin x} = \sum_{n=-\infty}^{+\infty} \mathrm{J}_n(\kappa)e^{-\mathrm{i}nx}$:

$$\mathrm{i}\hbar\frac{\partial}{\partial t}\rho_{ee} = \left[\frac{1}{2}\mathscr{E}\left(\mathrm{e}^{2\mathrm{i}(kz-\omega t)}+1\right) + E_{\text{signal}}\mathrm{e}^{\mathrm{i}(kz-\omega t)}\right]\sum_{n=-\infty}^{\infty}\mathrm{J}_n(\kappa)\mathrm{e}^{-\mathrm{i}n(kz-\omega t)}d_{eg}^{\star}r_{eg} + \text{c.c.} - 2\mathrm{i}\hbar\gamma_{\text{se}}\rho_{ee}, \tag{S7a}$$

$$\begin{aligned}\mathrm{i}\hbar\frac{\partial}{\partial t}r_{eg} &= \hbar\left[\delta - \frac{\partial}{\partial t}\kappa - E_{\text{signal}}(d_{ee}-d_{gg})\right]r_{eg} \\ &+ \left[\frac{1}{2}\mathscr{E}\left(\mathrm{e}^{2\mathrm{i}(kz-\omega t)}+1\right) + E_{\text{signal}}\mathrm{e}^{-\mathrm{i}(kz-\omega t)}\right]d_{eg}(2\rho_{ee}-1)\sum_{n=-\infty}^{+\infty}\mathrm{J}_n(\kappa)\mathrm{e}^{\mathrm{i}n(kz-\omega t)} - \mathrm{i}(\gamma_{\text{es}}+\gamma_{\text{dec}})r_{eg}.\end{aligned} \tag{S7b}$$

If $\Omega_R \ll \omega$, the oscillatory terms on the right-hand side of the above equations make negligible contributions. We neglect them under the rotating wave approximation, in which out of the infinite sums the only surviving terms correspond to $n = 0, 2$ if they are multiplied by the drive envelope $\mathscr{E}$, or correspond to $n = 1$ if they are multiplied by the signal field. We arrive at the equations (10) from the article.

To derive formula (12), we insert Eq. (1) into the right-hand side of Eq. (4). Performing all the derivatives, we obtain

$$\begin{aligned}-\frac{\partial^2}{\partial z^2}E + \frac{1}{c^2}\frac{\partial^2}{\partial t^2}E &= -\frac{\partial^2}{\partial z^2}E_{\text{signal}} + \frac{1}{c^2}\frac{\partial^2}{\partial t^2}E_{\text{signal}} \\ &+ \frac{1}{2}\left(-\frac{\partial^2}{\partial z^2} + \frac{1}{c^2}\frac{\partial^2}{\partial t^2} - 2\mathrm{i}k\frac{\partial}{\partial z} + 2\mathrm{i}\frac{\omega}{c^2}\frac{\partial}{\partial t} + k^2 - \frac{\omega^2}{c^2}\right)\mathscr{E}\left(e^{\mathrm{i}(kz-\omega t)} - \mathrm{e}^{-\mathrm{i}(kz-\omega t)}\right).\end{aligned} \tag{S8}$$

Clearly, the slowly varying terms of the expression correspond to the signal, while the terms related to the drive envelope are multiplied by rapidly oscillating factors.

Let us now investigate the right-hand-side of the wave equation with the second time derivative of polarization. The fastest method is to apply the derivatives to the form of polarization given by Eq. (11). We introduce the symbol

$$R = \sum_{n=-\infty}^{\infty} \mathrm{J}_n(\kappa)\mathrm{e}^{-\mathrm{i}(n-1)(kz-\omega t)}. \tag{S9}$$

The second time derivative of the polarization is

$$\frac{\partial^2}{\partial t^2}P = N\left[(d_{ee}-d_{gg})\frac{\partial^2}{\partial t^2}\rho_{ee} + 2\frac{\partial^2}{\partial t^2}\mathrm{Re}(d_{eg}^{\star}r_{eg}R)\right]. \tag{S10}$$

The second term in the bracket reads

$$\begin{aligned}\frac{\partial^2}{\partial t^2}(d_{eg}^{\star}r_{eg}R) &= d_{eg}^{\star}\left(R\frac{\partial^2}{\partial t^2}r_{eg} + 2\frac{\partial}{\partial t}r_{eg}\frac{\partial}{\partial t}R + r_{eg}\frac{\partial^2}{\partial t^2}R\right)\\ &= \sum_{n=-\infty}^{\infty}\left[\left(\frac{\partial^2}{\partial t^2}r_{eg} + 2\frac{\partial}{\partial t}r_{eg}\left(\mathrm{J}_n'(\kappa)\frac{\partial}{\partial t}\kappa + \mathrm{i}(n-1)\omega\mathrm{J}_n(\kappa)\right) + r_{eg}\left(\mathrm{J}_n''(\kappa)\left(\frac{\partial}{\partial t}\kappa\right)^2 + \mathrm{J}_n'(\kappa)\frac{\partial^2}{\partial t^2}\kappa\right.\right.\right.\\ &\quad\left.\left.\left. + 2\mathrm{i}(n-1)\omega\mathrm{J}_n'(\kappa)\frac{\partial}{\partial t}\kappa + [\mathrm{i}(n-1)\omega]^2\mathrm{J}_n(\kappa)\right)\right)d_{eg}^{\star}\mathrm{e}^{-\mathrm{i}(n-1)(kz-\omega t)}\right],\end{aligned} \tag{S11}$$

where we have explicitly inserted the first and second derivatives of R in accordance with Eq. (S9). We insert this result back into Eq. (S10).

Now, in analogy with the procedure conducted for the left-hand side (S8) of the wave equation, we can separate on the right-hand-side terms proportional to different powers of the oscillating factor $\exp[i(kz-\omega t)]$. In the rotating wave approximation, we neglect powers other than 0 and 1, as only these two are present on the left-hand side given by Eq. (S8). Now, we make the important assumption that any cross-talk between the slowly varying terms and those oscillating at the frequency ω can be neglected: The polarization terms oscillating at ω are coupled to the drive, while the slowly varying terms act as the source for the signal. This assumption is valid if $\left|\frac{\partial\mathscr{E}_{\mathrm{signal}}}{\partial t}\right| \ll \omega\mathscr{E}_{\mathrm{signal}}$ and similarly for the slowly varying part of the polarization. As a result, we can separate two wave equations, describing respectively the dynamics of the drive and of the signal. However, we assume the drive to be strong enough not to be affected by the coupling to the medium. The only relevant field equation is therefore the one for the drive, given by (12). On its right-hand side appear the terms corresponding to $n=1$ in Eq. (S11).

S3 Numerical approach

The solution of the coupled set of Bloch–Maxwell equations (10, 12) can be found numerically.

The wave equation has the form

$$-\frac{\partial^2}{\partial z^2}f(z,t) + \frac{1}{c^2}\frac{\partial^2}{\partial t^2}f(z,t) = s(z,t), \tag{S12}$$

where f is the function we wish to find, s is a source term, and c is the speed of the envelope in vacuum. Introducing discretization, we end up with a time-space grid of evenly distributed points (z_j,t_i) with respective spatial and temporal steps Δz and Δt. Hence, for the space variable, we have $z_j = z_0 + j\Delta z$, where $j \in [0,1,\ldots,N_L]$, and N_L is the number of the point at the end of the sample of length L, so $N_L\Delta z = L$. Similarly, $t_i = t_0 + i\Delta t$ for $i \in [0,1,\ldots]$ but with no upper bound. For convenience, we denote values of the functions at grid points $f(z_j,t_i) \equiv f_{i,j}$ and $s(z_j,t_i) \equiv s_{i,j}$. By the solution at time t_{i+1}, we understand the set of values $\{f_{i+1,j}\}$ for all j, and hence we have to find an expression that depends only on the previously calculated values. This can be done by expressing the second-order derivatives by the three-point (midpoint) formula[1]. Because we solve a second-order partial differential equation, two initial conditions for each space point are required. We introduce values $f_{j,0}$, $s_{j,0}$ and velocities $\partial f_{j,0}/\partial t \equiv g_j$ for the initial time t_0. To reach an accuracy on the order of $(\Delta t)^2$, we express $f_{1,j}$ as[2]

$$f_{1,j} = \frac{1}{2}\eta^2(f_{0,j-1}+f_{0,j+1}) + (1-\eta^2)f_{0,j} + \Delta t g_j + \frac{1}{2}c^2(\Delta t)^2 s_{0,j}, \quad \text{for } j \neq 0, N_L, \tag{S13}$$

and we find

$$f_{i+1,j} = \eta^2(f_{i,j+1}+f_{i,j-1}) + 2(1-\eta^2)f_{i,j} - f_{i-1,j} + c^2(\Delta t)^2 s_{i,j}, \quad \text{for } i>0, j\neq 0, N_L. \tag{S14}$$

Obviously, the foregoing expressions are not valid at the ends of the sample. There, we apply transparent boundary conditions[3] for the radiation exiting the sample (for $j = 0, N_L$):

$$f_{1,0/N_L} = \eta^2 f_{0,1/N_L-1} + (1-\eta^2) f_{0,0/N_L} + (1-\eta)\Delta t g_{0/N_L}, \tag{S15a}$$

$$f_{i+1,0/N_L} = \frac{2\eta^2 f_{i,1/N_L-1} + 2(1-\eta^2) f_{i,0/N_L} + (\eta-1) f_{i-1,0/N_L}}{1+\eta}, \tag{S15b}$$

where we assumed no sources at the ends of the sample. In addition, we have introduced the parameter $\eta = c\Delta t/\Delta z$, the so-called Courant number first described in Ref. 4, whose value is crucial for numerical stability. In our case, $\eta = 1$ corresponds to the so-called "magic step"[5] and leads to a solution that is not affected by the numerical dispersion problem.

The source term $s(z,t)$ corresponds to the solution of the Bloch Eqs. (10). The latter is a set of first-order differential equations of one variable for each point in space. At each time step, we calculate the solution using the Python build-in method *odeint* from the *scipy.integrate* library. It is a RK4 method, snd so the order of accuracy is $(\Delta t)^4$. This high level of accuracy is required because the source term is eventually differentiated twice, reducing the accuracy to the order of $(\Delta t)^2$. The differentiation is performed using the three-point (midpoint) method.

Our solver is written in Python 2.7, where two sets of equations (S14, S15) were implemented. A comparison between Eqs. (12) and (S12) reveals that $f \equiv E_{\text{signal}}$, and s is the right-hand side of Eq. (12). The solver is available on a public repository[6]. We draw the readers attention to another existing solver[7].

S4 Lithium hydride example

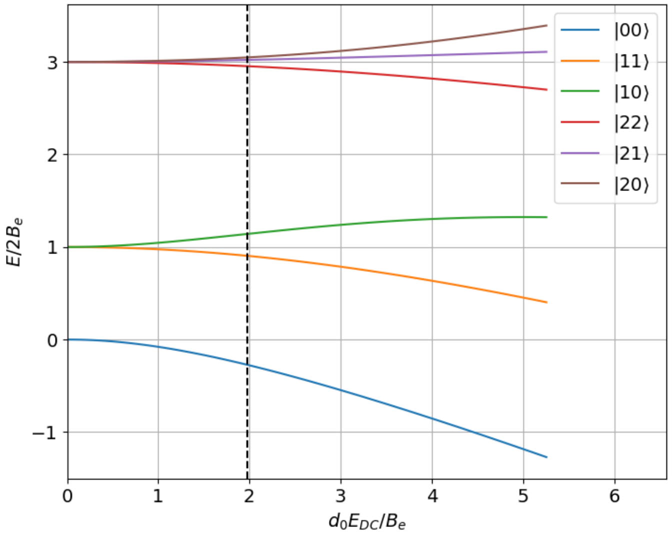

Figure S2. Energies of the rotational states in the ground electronic and vibrational state of the LiH molecule, as a function of electric field. The dashed line represents $E_{DC} = 150$ kV/cm. Levels represented by the blue and green lines were selected for the calculations in the main text.

To perform calculations based on a real system we have chosen the LiH molecule in ground state $X^1\Sigma^+$. We investigate its rotational states $|NM\rangle$. Since in the ground state the electronic angular momentum is zero, N and M denote the rotational angular momentum of the molecule and its projection. The permanent electric dipole moment in the molecule's frame is $d_0 = 5.88$ D[8] and the rotational constant $B_e = 7.513$ cm^{-1}[9]. In general, the orientation of a molecule in the gaseous ensemble is random and as the result, in the lab frame, the average dipole moment cancels out. To distinguish one of the directions (e.g. z-axis in the lab frame) and orient molecules we may apply an additional, constant electric field E_{DC}. This results in the DC

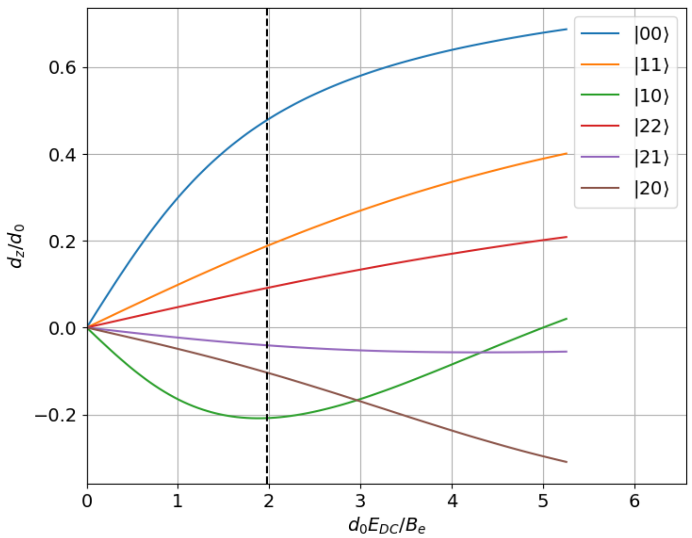

Figure S3. Projections of the permanent dipole moments in different rotational states as functions of applied electric field. The dashed line represents the amplitude $E_{DC} = 150$ kV/cm. The blue and green curves represent results for the states selected for the calculations in the main text.

Stark effect which leads to mixing of rotational states. The coupling matrix elements between states $|NM\rangle$ and $|N'M'\rangle$ read[10]

$$V_{NM;N'M'} = -E_{DC}d_0\sqrt{\frac{4\pi}{3}}\,\langle NM|\,\mathrm{Y}_{10}\,|N'M'\rangle\,, \tag{S16}$$

where the spherical harmonic Y_{10} has been used. The $3-j$ Wigner symbols may now be used to evaluate the transition elements. As a result, the following matrix representation of the Hamiltonian is obtained

$$H_{\text{stark}} = \begin{array}{c} \\ |00\rangle \\ |10\rangle \\ |20\rangle \\ |30\rangle \\ |11\rangle \\ |21\rangle \\ |31\rangle \\ |22\rangle \\ |32\rangle \\ |33\rangle \end{array} \begin{array}{c} \begin{array}{cccccccccc} |00\rangle & |10\rangle & |20\rangle & |30\rangle & |11\rangle & |21\rangle & |31\rangle & |22\rangle & |32\rangle & |33\rangle \end{array} \\ \left[\begin{array}{cccccccccc} 0 & E_{DC}d_1 & 0 & 0 & 0 & 0 & 0 & 0 & 0 & 0 \\ E_{DC}d_1 & 2B_e & E_{DC}d_3 & 0 & 0 & 0 & 0 & 0 & 0 & 0 \\ 0 & E_{DC}d_3 & 6B_e & E_{DC}d_6 & 0 & 0 & 0 & 0 & 0 & 0 \\ 0 & 0 & E_{DC}d_6 & 12B_e & 0 & 0 & 0 & 0 & 0 & 0 \\ 0 & 0 & 0 & 0 & 2B_e & E_{DC}d_2 & 0 & 0 & 0 & 0 \\ 0 & 0 & 0 & 0 & E_{DC}d_2 & 6B_e & E_{DC}d_5 & 0 & 0 & 0 \\ 0 & 0 & 0 & 0 & 0 & E_{DC}d_5 & 12B_e & 0 & 0 & 0 \\ 0 & 0 & 0 & 0 & 0 & 0 & 0 & 6B_e & E_{DC}d_4 & 0 \\ 0 & 0 & 0 & 0 & 0 & 0 & 0 & E_{DC}d_4 & 12B_e & 0 \\ 0 & 0 & 0 & 0 & 0 & 0 & 0 & 0 & 0 & 12B_e \end{array}\right] \end{array}, \tag{S17}$$

where $d_1 = -\frac{1}{\sqrt{3}}d_0$, $d_2 = -\frac{1}{\sqrt{5}}d_0$, $d_3 = -\frac{2}{\sqrt{15}}d_0$, $d_4 = -\frac{1}{\sqrt{7}}d_0$, $d_5 = -\frac{2\sqrt{2}}{\sqrt{35}}d_0$, $d_6 = -\frac{3}{\sqrt{35}}d_0$.

A diagonalization of the presented Hamiltonian allows us to find new eigenstates and eigenenergies of the system for different values of the DC electric field. The new eigenstates are superpositions of the original states $|NM\rangle$, and for the studied range of fields each eigenstate has one dominant contribution. The eigenenergies are presented in Fig. S2 for the amplitude E_{DC} in the range between 0 and 400 kV/cm. The label corresponds to the state $|NM\rangle$ whose contribution to the eigenstate dominates. As can be seen, the energy gap between the levels of interest grows with the electric field in the considered range.

The new eigenstates are characterized with a nonzero permanent electric dipole moment in the lab frame, oriented along the z-axis (the direction of the E_{DC}) as presented in Fig. S3. The black dashed line indicates the field amplitude used in the main text. Additionally, transition dipole moments between pairs of states can be induced. In Fig. S4 we present all components of the transition dipole moments between the ground state to a set of possible excited states. As expected, for $M = M' = 0$ the transition dipole moments are parallel to the z-axis while transitions between levels with $M' \neq 0$ correspond to dipoles in

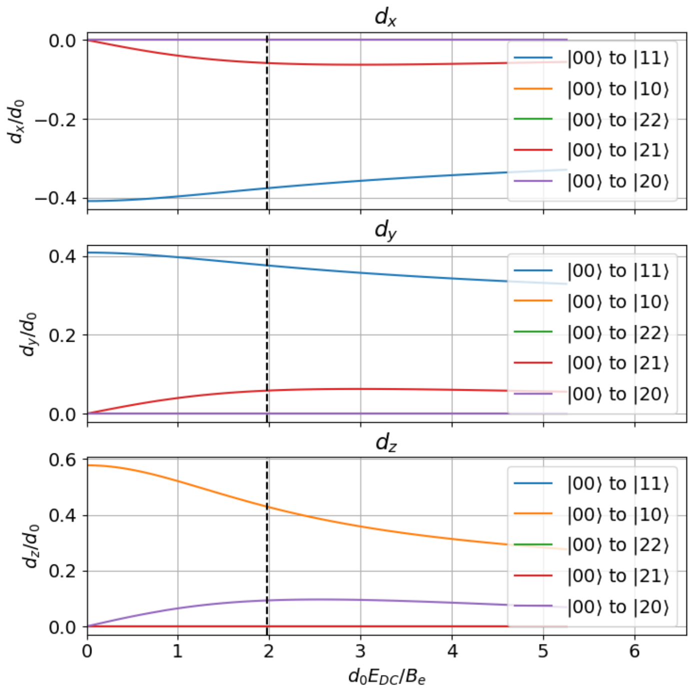

Figure S4. Projections on the laboratory frame axes of the transition electric dipole moments for transitions between the ground and selected excited states. The dashed line represents the amplitude $E_{DC} = 150$ kV/cm. The orange curve representes the results for the pair of states selected for the calculations in the main text.

oriented in the *xy* plane. These dipole moments decrease with the growing field due to the increasing contribution of states different than the dominant one.

References

1. Burden, R. L. & Faires, J. D. *Numerical Analysis* (Brooks/Cole, Cengage Learning, 2011).
2. Oliver, P. J. Numerical analysis lecture notes. http://www-users.math.umn.edu/~olver/num_/lnp.pdf (2008). [Online; accessed 29 July 2020].
3. Ionescu, D.-C. & Igel, H. Transparent boundary conditions for wave propagation on unbounded domains. In *International Conference on Computational Science*, 807–816 (Springer, 2003).
4. Courant, R., Friedrichs, K. & Lewy, H. On the partial difference equations of mathematical physics. *IBM journal Res. Dev.* **11**, 215–234 (1967).
5. Min, M. & Teng, C. The instability of the yee scheme for the "magic time step". *J. Comput. Phys.* **166**, 418–424 (2001).
6. Gładysz, P. Bloch–Maxwell simulation software. https://github.com/gladysz-piotr/Low-frequency_propagation.git (2020). [Online; accessed 29 July 2020].
7. Riesch, M. & Jirauschek, C. mbsolve: An open-source solver tool for the maxwell-bloch equations. *arXiv preprint arXiv:2005.05412* (2020).
8. Dagdigian, P. J., Wilcomb, B. E. & Alexander, M. H. Lih state-to-state rotationally inelastic cross sections in collisions with hcl and dcl. *The J. Chem. Phys.* **71**, 1670–1682 (1979).
9. Irikura, K. K. Experimental vibrational zero-point energies: Diatomic molecules. *J. physical chemical reference data* **36**, 389–397 (2007).
10. Krems, R. V. *Molecules in electromagnetic fields: from ultracold physics to controlled chemistry* (John Wiley & Sons, 2018).
